# Fitness information-seeking behavior among female university students: A qualitative study

**DOI:** 10.1371/journal.pone.0237735

**Published:** 2020-08-17

**Authors:** Soraya Jalali, Mahrokh Keshvari, Mohammad Reza Soleymani

**Affiliations:** 1 Department of Medical Library and Information Sciences, School of Management and Medical Information Sciences, Isfahan University of Medical Sciences, Isfahan, Iran; 2 Nursing and Midwifery Care Research Center, School of Nursing and Midwifery, Isfahan University of Medical Sciences, Isfahan, Iran; 3 Health Information Technology Research Center, Isfahan University of Medical Sciences, Isfahan, Iran; Iran University of Medical Sciences, ISLAMIC REPUBLIC OF IRAN

## Abstract

Obsession with the physique and appearance is a by-product of consumer societies. As such, fitness and slimming have now become major concerns of Iranian females. This study endeavors to elaborate on information-seeking behaviors among female students of Isfahan University of Medical Sciences concerning fitness. This study conducted in 2018, employs a qualitative approach using conventional content analysis. The research population includes female students of Isfahan University of Medical Sciences, 16 of whom were selected with use of purposive sampling. Data were collected through face-to-face semi-structured interviews and their trustworthiness was confirmed by the criteria of ‘credibility’, ‘confirmability’, ‘dependability’, and ‘transferability’ proposed by Guba and Lincoln. Results reveal four sub-categories and nineteen codes on information-seeking behavior. Sub-categories and codes including information-seeking motivations (achieving physical health, physical appearance, social acceptability, self-confidence, family and friends’ pressure) information resources (electronic information resources, social media, printed information resources, doctors and nutritionists, family and friends, traditional & Islamic medicine, radio and TV), information validation (asking the doctors and specialists, matching the information with scientific references, consulting with friends and relatives) and obstacles to seeking information (lack of time, high costs, distrust, access limitation). by increasing the students’ informational and media literacy, providing authentic and low-cost online resources of information and specialized TV programs, the damages rooted in using invalid information resources concerning fitness can be substantially diminished.

## Introduction

The Health information-seeking behavior involves the methods by which individuals consciously search diverse resources for information regarding their health, the risks, diseases, and manners of health preservation [1]. In other words, health information-seeking behavior is a purposive and active behavior for fulfilling an informational need. As an outcome, the information is obtained from different media and resources and is thereafter used. Such behavior represents the way through which individuals search, find, and apply the information related to a disease [2]. The findings of some studies related to the information-seeking behavior demonstrate that people use more accessible resources, such as the Internet, for seeking health-related information [3]. Fitness is one of the topics on which individuals', particularly the youth, are interested in acquiring information. Wang et al. [4] realized in their research that the health information-seeking behavior in people is affected by four categories of psychological, instrumental, contextual, and demographic factors.

In recent years, attaining fitness and an ideal weight has become matter of grave concern to the youth, particularly the girls; As such, diet, exercise, and bodybuilding exercises have interwoven into their daily routines to achieve a desired physical appearance. The modern society has transformed the physical image into a social phenomenon with cultural and symbolic values [5]. Body image is the way individuals see their body and the degree to which they consider themselves attractive [6]. By approaching adolescence and youth, individuals increasingly focus on their appearance and physical attractiveness [7]. Most of the young girls and boys are preoccupied with their body image [8]. They spend most of their time with their peers discussing their appearance and ways to attract the opposite sex and imitate celebrities [9]. The excessive preoccupation of young adults with beauty and fitness contributes to their engagement in rather extreme behaviors to adopt cultural patterns of beauty that are advertised a variety of media and on the Internet [10]. In the past, individuals used to follow more conventional methods of beauty and fitness, but the emergence of media and information resources has changed their attitudes and behaviors. Information-seeking behavior is the way individuals search and use information related to physical and mental health, health threats, and health promotion practices [11].

The findings of some studies showed that in recent years in most societies, including Iran, young girls are more obsessed with their body and physical appearance since body for them conveys a sense of identity [10]. It seems that the important reason for females' obsession is autophobia and a negative body image [12]. The findings of some studies revealed that body management, has a positive and significant relationship with media consumption, social acceptance of the body, and socioeconomic status; though, religiosity and body management are in an inverse relationship [13]. Undoubtedly, TV has a major role in disseminating health information and individuals obtain the majority of their health-related information by watching TV [14].

In addition to television, owing to a wide range of capacities as well as high flexibility in providing health information, the Internet inspires students to seek health information and promote their health [15]. Findings of some studies showed that more than 90% of adolescents receive their health-related information about fitness, sexual health, and nutrition from social networks [16]. Jacob et al. [3] found that the Internet has become the single most important source of information about health. Literature suggests that despite the importance of health information, particularly concerning fitness, not many studies have investigated the fitness information-seeking behaviors. The present study investigates the female students’ information-seeking behavior about fitness. Given the high rates of obesity in Iran, especially the severe rise in the obesity prevalence among Iranian women, as well as the intense inclination of women in the province of Isfahan toward fitness, the findings of this study can provide essential information for Iran’s health policymakers to eliminate the barriers for seeking fitness-related information and reduce the consequences of using invalid information [17].

## Materials and methods

The qualitative study using conventional content analysis was carried out from February to July 2018. The participants were female students of different schools of Isfahan University of Medical Sciences. Participants are identified and selected using purposive sampling method. Also, snowball sampling was used for identifying respondents armored with rich information relevant to the study. The Inclusion criteria were the tendency to participate and intending to be fit in the present or the past. The exclusion criterion was withdrawal from participation. For data collection, the researcher went to the faculties, selected the participants, explained the purpose of the study to them, and after obtaining their consent for participation, the time and place of the interviews were arranged.

Semi-structured interviews were used to collect data. The interview questions were developed by the research team (S1 File) and one of the trained researchers conducted the interviews. Before starting the formal interviews, a practice interview was carried out under the surveillance of the main researcher. To foster trust between both parties, the interviews were started by asking open-ended questions like “Please talk about what you have done to attain fitness so far?”, additionally, to clarify the participants’ responses, they were asked to give real-life examples of their statements. The saturation point was reached at 16 face-to-face semi-structured interviews. The interviews were recorded by a voice recorder. Each interview lasted about 30 to 60 minutes depending on the participant’s willingness and her information on the research material.

The gathered data were analyzed by conventional content analysis by MAXQDA version 10. Based on the research stages proposed by Graneheim and Lundman [18], data analysis was performed continuously and simultaneously with data collection. In the first stage, the sentences and paragraphs that included concepts related to the subject of the study were chosen as semantic units. The initial codes were extracted by converting the semantic units into more brief expressions. At this stage, 164 initial codes were extracted, which were subsequently reviewed and compared. Similar cases were then combined into a single topic. Afterwards, 19 codes were extracted. In the next stage, the related codes were categorized. Ultimately, the concept and hidden content within the data were extracted as the main category by a re-evaluation of the codes and levels.

The four trustworthiness criteria of credibility, confirmability, dependability, and transferability suggested by Lincoln and Guba were used for assessing the quality of the study [19]. Credibility was ensured by the researcher’s prolonged involvement in the project and ongoing interaction with the participants, which in turn facilitated gaining the participants’ trust and a better understanding of their experiences. To ensure the credibility of the research, the findings were returned to some participants for further comments. The dependability of the research was ensured by the implementation of interviews as soon as possible, accurate recording of all stages of the research, and providing the participants with equal situations. To promote the transferability of the findings, we tried to select participants who were diverse in terms of faculty, major, and degree. Finally, parts of the text of the interviews, along with the codes and categories extracted for evaluation, were provided to three experts outside the research team to confirm the correctness of the given data.

To honor ethical considerations, the study was approved by the Ethics Committee of Isfahan University of Medical Sciences before being conducted. Before doing the research, the participants were briefed on the research purposes, data privacy, recording the interviews, and on their authority to attend or leave the interview and their written consent to participate and the recorded interviews. To respect the participants’ privacy, every individual was assigned a code and the data were reported anonymously.

## Results

Sixteen female students participated in this research. They are described in Table 1 (i.e. their faculty, major and degree).

**Table 1:** Participants' characteristics.

Participant	Faculty	Major	Degree
P1	Management and Medical Information Sciences	Medical Librarianship and Information Sciences	Postgraduate
P2	Pharmacy and Pharmaceutical Sciences	Medicinal Chemistry	Postgraduate
P3	Medicine	Medicine	Undergraduate
P4	Nursing and Midwifery	Nursing	Undergraduate
P5	Health	Occupational Health and Safety Eng.	Undergraduate
P6	Rehabilitation Sciences	Physiotherapy	Undergraduate
P7	Management and Medical Information Sciences	Healthcare Services Management	Undergraduate
P8	Health	Occupational Health and Safety Eng.	Postgraduate
P9	Health	Environmental Health Eng.	Undergraduate
P10	Rehabilitation Sciences	Physiotherapy	Undergraduate
P11	Rehabilitation Sciences	Physiotherapy	Undergraduate
P12	Rehabilitation Sciences	Audiology	Undergraduate
P13	Rehabilitation Sciences	Speech Therapy	Undergraduate
P14	Nursing and Midwifery	Nursing	Undergraduate
P15	Dentistry	Dentistry	Undergraduate
P16	Dentistry	Dentistry	Undergraduate

Based on the content analysis of the interviews regarding fitness information-seeking behavior, four categories were extracted including information-seeking motivations, information resources, information validation, and the obstacles for information-seeking (Table 2).

**Table 2:** Category, sub-categories, and codes related to the fitness information-seeking behavior.

Category	Sub-categories	Codes
Health Information Seeking Behavior	Information seeking motivations	Physical health
		Beauty
		Social acceptability
		Gaining self-confidence
		Family and peer-pressure
	Information resources	Electronic information resources
		Social media
		Printed information resources
		Physicians and nutrition specialists
		Family and friends
		Traditional & Islamic medicine
		Radio and TV
	Information validation	Enquiring from the physicians and specialists
		Conformity with scientific resources
		Consulting friends and acquaintances
	Information-seeking barriers	Shortage of time
		High costs
		Lack of trust
		Access limitation

### A. Information-seeking motivations

The participants’ experiences indicated five reasons for seeking information on fitness, which include gaining physical health, beauty, social acceptance, self-confidence, and family and peer-pressure.

#### Physical health

Most of the interviewees noted that physical health is an important motivation for being fit and gaining information about it. They recognized obesity as a negative factor in health and life expectancy, which probably entails different diseases and even high mortality rates. Participant 4 said: *“When I go to the hospital and my colleagues tell me that a patient who had diabetes and hypertension has died*, *it really affects me because obesity is linked to diabetes and hypertension”*. Furthermore, some participants were of the opinion that much like obesity, being underweight or excessively thin could, in addition to beauty issues, lead to different diseases, especially loss of muscle mass and inability to do daily tasks. *“If you are skinny*, *you will have muscle weakness or will not be able to do a lot of things because of physical feebleness*. *You will get tired or feel sleepy too soon” (p*. *11)*.

#### Beauty

From the participants’ view, cosmetic beauty is more emphasized than natural beauty in today’s world. Besides, they believed that physical fitness is considered as one of the important measures of beauty, particularly in females. This matter has triggered them to look for information on how to lose weight faster to gain fitness. *“My primary motivation is to look beautiful and to gain confidence as a result*. *I think these two factors are enough for me” (p*. *12)*. Some others believed that if they were fit, they could wear better attire, which would bolster their beauty. *“It is great when the clothes you like*, *fit you well and make you look more beautiful wearing them*. *What more can a girl want other than beauty*?*” (p*.*13)*.

#### Social acceptance

Based on the participants’ experiences, nowadays beauty and fitness are two important benchmarks of social evaluation for individuals. Beautiful and fit people, compared to the others, are placed in a better social position. They considered beauty and fitness not as a personal affair, but as a social issue that involves different benefits and consequences such as gaining acceptance and suitable social positions. *“Truth be told*, *appearance is very important in our society*. *Unfortunately*, *no one pays attention to other good attributes that you have*. *The first thing they notice is how you look*. *If you have a good appearance*, *you will attain good social positions” (p*. *6)*.

#### Gaining self-confidence

The results of the research illustrated that one of the participants’ motivations for acquiring information regarding fitness is the confidence they receive by being physically fit and beautiful. They are convinced that their mental image of the body affects their behavior and conduct; therefore, being unsatisfied with their physical state causes their loss of self-confidence. Consequently, they constantly attempt to obtain fitness via different routes such as going on diets, exercising, and so on. In this regard, interviewee number 6 said: *“I like my appearance to be in a way that others find it nice because then I shall feel confident”*.

#### Family and peer-pressure

According to some of the participants, one of the reasons they began thinking about fitness and looking for avenues to reach it is the pressure applied by their family and friends. They revealed that they constantly receive different comments about their appearance and body from their friends, which has led to their dissatisfaction with their bodies. Moreover, they feel pressure from their family about their diets and exercise routines to become fit. Interviewee number 15 commented: *“My friends and acquaintances are my biggest motivating forces for finding information about gaining fitness*. *My best friend always pays attention to her fitness*. *She exercises and encourages me to do the same”*. Likewise, interviewee number 13 said: *“My mother always asks me to be fit*, *eat less*, *and eat healthy food”*.

### B. Information resources

The findings attained through analyzing the interviews demonstrated that the participants achieve fitness-related information from various channels as per the following:

#### Electronic information resources

Most of the participants used electronic information resources to reach information about health and the ways to gain fitness. In particular, they utilized the websites of the physicians and fitness specialists as well as online databases. They believed that the Internet is the most accessible and affordable resource for obtaining the newest information in any field. *“I use the Internet merely to visit the websites of Dr*. *Kermani and some other famous specialist physicians in the field of fitness” (p*. *5)*. Also, participant number 16 noted: *“The Internet is the most accessible resource among others*. *I search for many things on the Internet*. *For instance*, *I search about diets*, *helpful ingredients for losing weight*, *the proper wait time between meals*, *etc*.*”*

#### Social media

One of the other resources of information used by the majority of the participants is social media, both in the application or web-based forms. Since an abundance of expert information is constantly available in groups, channels, and specialized pages on social media, the participants recognized them as the best means for receiving various pieces of training and updating their information on different aspects of health issues including weight loss diets and appropriate exercises for gaining fitness. *“I use specialized Instagram pages that share exercises for trimming the hips*. *They also share the related images and videos” (p*. *13)*. Additionally, participant number 7 mentioned: *“Some Telegram channels share information about fattening food or spot reduction exercises*, *which are very useful and I used them a lot”*.

#### Printed information resources

The experiences of some participants indicated the application of printed resources of information, in particular related to healthy diets for fitness. Since they had access to specialized diet books in the libraries of the university, they could benefit from these resources. The fitness sections of popular magazines were another resource used by the participants for this purpose. Participant number 2 said: *“I read a lot of diet books*. *For instance*, *there was a book about the principles of diets*, *which contained information such as the number of calories consumed by eating each type of food*. *I also read magazines that publish interesting content such as interviews with people who have lost weight”*. Also, participant number 6 said *“In my opinion*, *books are more appropriate than the Internet for gaining information about health because we know who the author is and whether he or she is an expert”*.

#### Physicians and nutrition specialists

Most of the participants regarded physicians and nutrition specialists as the most trustworthy resources for obtaining health information, including the ways to attain fitness. They believed that given physician nutrition specialists possess sufficient specialization and renown, in addition to providing scientific methods for beauty and fitness, they can assist in controlling the side effects of diets or the underlying diseases. *“Due to the trust I have in physicians and specialists*, *I met a physician nutrition specialist to lose weight*. *I tried to do all the tasks related to my fitness precisely according to the specialist’s recommendation” (p*. *10)*. Similarly, interviewee number 9 said: *“Considering the unique conditions of each person*, *public prescriptions may not fulfill their needs*. *I usually try to receive my fitness plan based on my conditions from a specialist after being examined”*.

#### Family and friends

According to the participants, one of the significant resources for finding information is using the guidance and experiences of friends and family members, especially those with prior experience in achieving their fitness goals. They believed that the individuals who were able to attain fitness in similar conditions can be considered as a reliable and simultaneously free resource for obtaining information. *“My father is an athlete and is very fit because of exercising*. *I always try to follow his advice” (p*. *14)*. Participant number 8 said: *“Since I am currently studying in a medical sciences university*, *I have friends who are nutrition specialists*. *I usually use their help in this regard”*.

#### Traditional and Islamic medicine

The other resource used by a few of our participants for obtaining information on different methods for improving their fitness is traditional and Islamic medicine. They were convinced that the approaches provided by traditional and Islamic medicine resources have fewer or no side effects because chemical substances are not used in traditional and Islamic medicine. As a result, instead of visiting a physician or nutrition specialist, some participants used herbal medicine and supplements shops and employ herbal remedies and infusions recommended by traditional medicine resources. In this regard, interviewee number 8 said: *“The information we gain from traditional medicine positively affects our fitness as well as other health aspects such as digestion”*.

#### Radio and television

Radio and TV programs, as well as the advertisements on national television and radio, are the other resources that some participants used for acquiring fitness-related information. They stated that the content on radio and TV is reliable since the Ministry of Health and Medical Education reviews and then approves all the programs and advertisements. *“I gain most of my information from the physicians and specialists that do interviews on television or radio as they are more reliable” (p*. *12)*.

### C. Information validation

The validity and accuracy of the information gained from different channels and resources was one of the main concerns of the interviewees, particularly the information about weight loss and fitness where people are more susceptible to being deceived by fraudulent individuals or organizations. The findings from the interviews showed that the participants measure the validity of the information through the following ways:

#### Enquiring from physicians and specialists

Regarding health-related information, individuals always trust physicians and specialists. Almost all of the participants emphasized on assuring the validity of the gained information. They declared that to ensure the validity of the information obtained from social media and other resources, they would talk to an expert such as physicians, nutrition specialists, or even sports coaches, despite the high costs involved, limited time, and the difficulty of access. *“I ensure the validity of the obtained information by asking questions from qualified people such as specialists and sports coaches*. *Most of my current knowledge regarding fitness is gained from my track and field coach*. *If I face any new question or doubt any information*, *I would ask him” (p*. *14)*.

#### Conformity with scientific resources

One of the methods used by the participants for evaluating the quality and validity of the acquired information is cross-referencing it with authentic and scientific resources. They believed that most of the information comes from unknown resources and cannot be trusted; hence, they tried to cross-reference the information with known scientific resources such as specialized books or investigate the validity of their source. Participant number 1 said: *“When I find some information*, *I pay attention to its author to discern if it was written by a physician or specialist*. *Also when I discover a new approach*, *I search for its potential side effects and whether other resources confirm it as well”*. Furthermore, participant number 2 said: *“When I visit a website*, *I consider its owner or administrator*. *If they are authentic*, *for instance like the Iranian Society of Nutrition*, *I can use it with ease of mind”*.

#### Consulting friends and acquaintances

Sharing the obtained information with friends and acquaintances is one of the measures by which some participants gauged its validity and credibility. They stated that when they find a weight loss recommendation, they try to consult others or individuals who have had successful experiences in losing weight to get information about the possible side effects. Participant number 16 said: *“I have a cousin who exercises a lot and his friends have been doing so as well*, *for a long time*. *Sometimes I ask him about the accuracy of the information I have heard or received”*.

### D. Information-seeking barriers

The results of the interviews revealed that the participants encounter different barriers and hardships while seeking fitness-related information.

#### Shortage of time

One of the limitations that students encountered while seeking information was the shortage of time. They indicated that they do not have much time for obtaining fitness-related information from authentic resources. This is due to various reasons such as large amounts of assignments, crowded offices of physicians or specialists, and so on. Therefore, participants prefer to use the more accessible resources for this purpose. Participant number 9 said: *“The major limitation is the shortage of time*. *Since most of my time is spent in classes at university*, *I do not have enough time for physician or specialist appointments*. *Thus*, *I would rather use the information on social media”*.

#### High costs

The experiences of some participants demonstrated that despite placing great trust in physicians, dieticians, and nutrition specialists, they face many hardships in using such authentic resources due to financial inability and high medical tariffs. To make the matters worse, none of the insurance companies accepts the costs related to fitness. One of the participants stated: *“The cost of visiting a specialist is too high that I had to put it aside for now since covering the costs of my university and purchasing books are my number one priority” (p*. *3)*.

#### Lack of trust

One of the crucial challenges that the participants faced through seeking and applying fitness-related information is their lack of trust in the information. In particular, the information obtained from satellite TV channels, websites, and social media lacks any concrete resource. Therefore, the possible dangers of their application have created a great dilemma for the participants. Accordingly, interviewee number 2 said: *“In social media applications such as Telegram*, *you can never know who has published a certain content or whether they were qualified to do so or whether they knew anything about fitness at all*!*”*

#### Access limitation

According to the participants, despite the ever-increasing application of social media in different aspects of people’s lives, especially in the field of health, many of these mediums such as Telegram, Twitter, YouTube, and some others are blocked in Iran. Hence, the participants could not reach these resources of information without using unblocking applications. This issue has led to the deprivation of beneficial content related to fitness. In this regard, interviewee number 15 said: *“Many of the social networks and websites are blocked in Iran and we must use unblocking applications to access them*. *For example*, *educational content is abundant on YouTube*. *However*, *they are all blocked and out of access”*.

## Discussion

The current research was conducted to clarify the information-seeking behavior related to fitness in female students via a qualitative approach. According to the findings of the research, four categories of information-seeking motivations, information-seeking resources, information validation, and information-seeking barriers were extracted within the health information-seeking behavior of students.

The research findings suggest that female students are obsessed with their physical appearance and tend to seek reliable information about how they can attain or maintain fitness. Information need acts as the initiating and instigating point in the health information-seeking behavior of students. In Wilson’s opinion, information need is one of the secondary needs, which is derived from the primary psychological, cognitive, and affective needs [20]. Furthermore, the first stage of the Kuhlthau's model of the Information Search Process (ISP) is “initiation” in which the person realizes that some information is required to resolve a certain problem [21]. This stage is similar to Wilson's model of information-seeking behavior in which the person identifies a perceived information need within a certain environment [20]. The findings of some studies such as the research conducted by Haghighatiyan et al [5] and Sholeha et al [8] show that for several reasons, women are more concerned about their fitness. Participants in this study motivated by reasons like improving physical and mental health, gaining social acceptability and self-confidence or due to their friends’ pressure sought fitness information. The findings of the research by Riyahi et al. confirm that seeking health information for having a healthy lifestyle is among the participants most important information needs [22]. Given obesity contributes to joint pains and many other diseases, it is common for people and the youth, in particular, to try to lose weight and attain fitness. Abdolhosseini and Haghighatiyan argue that there is a significant relationship between attention-seeking and body improvement behaviors [10]. Although striving for beauty is intrinsic motivation, these days has been replaced by obsessive flaunting and body showoff. Mahmoudi and Mohadesi believe that overrated attention to physical beauty in society leaves people prone to fear, doubt, and lack of confidence [12]. Moreover, some studies have shown that there is a direct relationship between social acceptability, self-confidence, fitness, and body image [13, 23, 24]. The excessive attention of newly emerged media to physical beauty and appearance has fueled fears of being excluded, losing the opportunity of marriage, finding a job, and etc. among the youth.

The findings show that the participants use different formal and informal resources to obtain information about fitness and body management. Initiating the information-seeking behavior means the students’ deliberate act to search their required information from various resources concerning fitness. Such an act is performed to satisfy an information need regarding fitness, which is stated in Wilson’s model of information-seeking behavior as “active information-seeking” [20]. Findings of some studies prove that the Internet as a new source of information has become more popular in mainstream society at large and among the youth in particular for obtaining health-related information among other purposes [25–29]. Furthermore, some studies suggest the growing popularity of the Internet and social networks for seeking health-related information among people [16, 27, 30, 31] whereas Finny Rutten et al. maintain that despite the availability of health information over online networks, these resources are not used so frequently [32]. Nevertheless, it seems that due to the updated and comprehensive information available on the Internet, the ease of access, and the high costs of visiting doctors; most individuals despite trusting doctors and specialists, opt for electronic resources to obtain information.

According to the participants’ experiences, one of the major challenges in seeking information was the disparities between the information obtained from different resources and uncertainty of the information accuracy. Therefore, the participants try to validate the received information by asking doctors and specialists, seeking out scientific references for information, and asking friends and relatives. The verification behavior of the obtained information is similar to the “verifying” stage in the Ellis’ model of information-seeking behavior who believes that the authenticity of the obtained information must be ensured by a variety of methods before their practical application [33]. Nasrollahzadeh found that when people are not sure about the accuracy of obtained information, they share it with other people particularly their family members and friends [34]. Moreover, the findings some studies demonstrated that despite people’s approximate trust in the information obtained from Internet websites, they consult specialist physicians before applying them [27, 35]. Due to the importance and sensitivity of health information, participants tend to use such information carefully. They trust physicians more than other individuals and resources, and most people believe that “doctors know best”.

According to the research findings, the participants come across different obstacles in searching for fitness-related information like. Shortage of time, high costs of visiting doctors, disparity, and distrust of information, and limited access to websites and social networks. In his research, Nasrollahzadeh demonstrated that the participants pointed to the time limitations as one of the barriers to information-seeking behavior. Therefore, they prefer using oral resources since they require a lesser time investment [34]. Latifi et al. find that participants prefer to obtain their needed information from doctors; however, they refer to doctors less frequently since they are not able to afford it [36]. Additionally, Perzeski, in his research, considered the high costs of information resources as one of the important information-seeking barriers, which deters individuals from utilizing them due to financial inability [37]. In Iran, none of the insurance companies cover the costs of cosmetic and fitness services, so most people cannot afford to pay for doctor or nutritionist visits. Accordingly, despite distrust in free available information, individuals usually do not have any alternatives. Moreover, some social networks and websites are blocked and therefore, many users are deprived of potentially useful information.

### Limitations

One of the limitations of this study was the difficulty regarding identifying the students who have in the present or the past, experienced making attempts in way of their fitness. The researchers utilized the snowball sampling method in order to solve this issue. The other limitation was the relatively high information and media literacy of the students compared to common individuals, which barred the extension of the findings of this study to include the information-seeking behavior of the common people as well. This way, doing researches on the information-seeking behavior of other groups shall provide the ground for analysis in those regards.

## Conclusion

This study assessed the fitness-related behavioral information of female collegiate students using an exploratory approach. The results indicated that students attempted to obtain fitness with different motives and gathered information on how to achieve fitness from various sources but they evaluated the validity and accuracy of the data to some extent before putting them into practice. Given the importance of health information, especially fitness, the study focuses on improving people's behavior regarding obtaining health information. Thus, although the research findings cannot be generalized, policy actions may be provided including promoting the general public’s health literacy and media literacy, providing credible information on how to obtain fitness through formal and accessible sources of information and health treatment insurance coverage related to fitness. Furthermore, conducting studies regarding the health information requirements of individuals about fitness and the reasons for their trust or lack of trust in some information sources for obtaining health information can be beneficial.

## Supporting information

S1 File Interview guide. (PDF)

S2 File Dataset. (MX4)

## References

[1] Lambert SD, Loiselle CG. Health Information Seeking Behavior. Qualitative Health Research; 2007, 17(8): pp. 1006–19. 10.1177/1049732307305199 17928475

[2] Jung M. Determinants of health information-seeking behavior: implications for post-treatment cancer patients. Asian Pac J Cancer Prev 2014; 15(16): 6499–504. 10.7314/apjcp.2014.15.16.6499 25169477

[3] Jacobs W, Amuta AO, Jeon KC. Health information seeking in the digital age: An analysis of health information seeking behavior among US adults. Cogent Social Sciences. 2017;3(1):1302785.

[4] Wang X, Shi J, Kong H. Online Health Information Seeking: A Review and Meta-Analysis. Health Communication. 2020 Apr 16:1–3. 10.1080/10410236.2020.1748829 32290679

[5] Haghighatian M, Ansari E, Asgari N. Physical Fitness and Social Factors Case of Women in Isfahan. Women's Studies Sociological and Psychological, 2012; 10(4): 159–179. 10.22051/jwsps.2012.1437

[6] Donglikar CV. Body Image Concern: Social Media and Adolescents. Our Heritage. 2020 1 23;68(57):279–88.

[7] Barkhordari-Sharifabad M, Vaziri-Yazdi S, Barkhordari-Sharifabad M. The effect of teaching puberty health concepts on the basis of a health belief model for improving perceived body image of female adolescents: a quasi-experimental study. BMC Public Health. 2020 12;20(1):1–7. 10.1186/s12889-019-7969-5 32197594 PMC7083033

[8] Sholeha EP, Ayriza Y. The Effect of Body Images and Self-Esteem on Subjective Well-Being in Adolescents. International Journal of Multicultural and Multireligious Understanding. 2020 3 13;6(4):635–45.

[9] Devi N. Body Image, Peer Pressure and Self-Esteem among Adolescents. International Journal of Creative Research Thoughts (IJCRT). 2018 4; 6(2): 120–32.

[10] Abdolhoseyni A, Haghighatian M. Social Factors Affecting on Body Management in Youth and Young Girls and Boys in the City of Isfahan. Refahj. 2017; 17 (67):233–71.

[11] Lambert SD, Loiselle CG, Macdonald ME. An in-depth exploration of information-seeking behavior among individuals with cancer: part 1: understanding differential patterns of active information seeking. Cancer Nursing. 2009 1 1; 32(1):11–23. 10.1097/01.NCC.0000343372.24517.bd 19104197

[12] Mahmoodi Y, Mohadessi Ghilovaei H. A sociological study of body industry: Qualitative study of the causes and consequences of cosmetic surgery of women living in Tehran 1395. Women in Development & Politics, 2017; 15(4): 523–47.

[13] Fatehi AG, Ekhlasi E. Body Management and its relationship with social acceptance of body. Women's Strategic Studies, 2008; 11: 9–42.

[14] Zare A, Rahimi S, Soofi K. The study of the information seeking behavior of health literacy among students of Razi University of Kermanshah. Journal of Health Literacy. 2017; 2(2):63–72.

[15] Hassan S, Masoud O. Online health information seeking and health literacy among non-medical college students: gender differences. Journal of Public Health. 2020 3 9:1–7.

[16] Plaisime M, Robertson-James C, Mejia L, Núñez A, Wolf J, Reels S. Social Media and Teens: A Needs Assessment Exploring the Potential Role of Social Media in Promoting Health. Social Media+ Society. 2020 2;6(1):2056305119886025.

[17] GBD 2015 Obesity Collaborators. Health effects of overweight and obesity in 195 countries over 25 years. New England Journal of Medicine. 2017 7 6;377(1):13–27. 10.1056/NEJMoa1614362 28604169 PMC5477817

[18] Graneheim UH, Lundman B. Qualitative content analysis in nursing research: concepts, procedures and measures to achieve trustworthiness. Nurse education today. 2004; 24(2):105–12. 10.1016/j.nedt.2003.10.001 14769454

[19] Lincoln YS, Guba EG. Naturalistic inquiry. Sage; 1985.

[20] Wilson TD. Human information behavior. Informing Science. 2000;3(2):49–55.

[21] Kuhlthau CC. Inside the search process: Information seeking from the user's perspective. Journal of the American society for information science. 1991;42(5):361–71.

[22] Riahi A, Hariri N, Nooshinfard F. Study of health information needs and barriers to access among Afghan and Iraqi immigrants in Iran. Journal of North Khorasan University of Medical Sciences. 2016 1 10; 7(3):597–610.

[23] Jameel HT, Shamim F. Relationship of Self-confidence with self-body image of visually impaired children. Journal of Research in Psychology. 2019 4 7;1(1):9–11.

[24] Sajadian I. Girls' confidence in their fitness. Health and Development. 2012; 4: (28).

[25] Bigdeli Z, Hayati Z, Heidari GR, Jowkar T. Place of Internet in health information seeking behavior: Case of young Internet users in Shiraz. Human Info Interact. 2016; 3 (1).

[26] Sedrak MS, Soto-Perez-De-Celis E, Nelson RA, Liu J, Waring ME, Lane DS, et al Online Health Information–Seeking Among Older Women with Chronic Illness: Analysis of the Women’s Health Initiative. Journal of Medical Internet Research. 2020;22(4): e15906 10.2196/15906 32271152 PMC7319595

[27] Obasola OI, Agunbiade OM. Online health information seeking pattern among undergraduates in a Nigerian university. SAGE Open. 2016 3 8;6(1):2158244016635255.

[28] Kyriacou A, Sherratt C. Online health information–seeking behavior by endocrinology patients. Hormones. 2019 11 20:1–1. 10.1007/s42000-019-00159-9 PMC6978446 31749117

[29] Baji F, Haghighizadeh MH, Karimzadeh-Bardei A. Investigation of Online Health Information Seeking Behavior among University Students in Ahvaz City, Iran. Health Inf Manage 2019; 16(4): 197–202

[30] McLean SA, Jarman HK, Rodgers RF. How do “selfies” impact adolescents' well-being and body confidence? A narrative review. Psychology research and behavior management. 2019; 12:513 10.2147/PRBM.S177834 31372071 PMC6628890

[31] Franchina V, Coco GL. The influence of social media use on body image concerns. International Journal of Psychoanalysis and Education. 2018 6 20;10(1):5–14.

[32] Finney Rutten LJ, Blake KD, Greenberg-Worisek AJ, Allen SV, Moser RP, Hesse BW. Online Health Information Seeking Among US Adults: Measuring Progress toward a Healthy People 2020 Objective. Public Health Reports. 2019 11; 134(6):617–25. 10.1177/0033354919874074 31513756 PMC6832079

[33] Ellis D. Modeling the information-seeking patterns of academic researchers: A grounded theory approach. The Library Quarterly. 1993;63(4):469–86.

[34] Nasrollahzadeh S. Health information-seeking behavior of pregnant women: A grounded theory study. Human Information Interaction. 2015 3 10; 1(4):270–81.

[35] Jaks R, Baumann I, Juvalta S, Dratva J. Parental digital health information seeking behavior in Switzerland: a cross-sectional study. BMC public health. 2019 12;19(1):225 10.1186/s12889-019-6524-8 30791927 PMC6385444

[36] Latifi M, Barahmand N, Fahimnia F. Post-mastectomy Barriers for Information Seeking in Women with Breast Cancer. Health INF Manage 2017; 13(5): 326–32.

[37] Perzeski DM. Information-seeking behaviors of podiatric physicians. Journal of the American Podiatric Medical Association. 2012;102 (6): 451–62. 10.7547/1020451 23204196

