# Supplementary material for: Fitness information-seeking behavior among female university students: A qualitative study
Source: PLoS One. 2020 Aug 17;15(8):e0237735. doi: 10.1371/journal.pone.0237735 (PMC7430743; doi:10.1371/journal.pone.0237735)
Supplement: S1 File — (PDF) [file pone.0237735.s001.pdf]

Interview guide

The main question	The secondary question
1. How important is being fit to you? If it is important to you, what efforts have you made so far to fulfill it?	• The reasons of importance<br>• The type of actions you have taken to gain fitness.
2. How do you get information about fitness when you need it? In other words, how do you fulfill your information needs?	• The used information sources<br>• The reasons for trusting or not trusting the fitness information sources<br>• Evaluating the correctness of information
3. Can you easily get information about fitness in case of need? In other words, what challenges have you faced in the process of searching and using the information?	• Facilitators<br>• Limiters
